# Molecular Subtyping, Receptor Concordance, and Magee Score Assessment in Breast Carcinoma: An Immunohistochemical Study

**DOI:** 10.7759/cureus.113127

**Published:** 2026-07-21

**Authors:** Shrinivas B Somalwar, Prarthana Bhushan, Shailaja Prabhala

**Affiliations:** 1 Department of Pathology and Laboratory Medicine, All India Institute of Medical Sciences, Bibinagar, Hyderabad, IND; 2 Department of Medical Oncology, Nizam’s Institute of Medical Sciences, Hyderabad, IND

**Keywords:** breast carcinoma, er, her2/neu, immunohistochemistry, ki-67, luminal a, magee score, molecular subtyping, pr, receptor discordance

## Abstract

Background: Breast carcinoma is a heterogeneous disease characterized by variable expression of estrogen receptor (ER), progesterone receptor (PR), HER2/neu, and Ki-67, which influence molecular classification and therapeutic decision-making. This study evaluated the immunohistochemical profile of invasive breast carcinoma, assessed receptor concordance between primary tumors and corresponding metastatic deposits, and estimated recurrence risk using Magee score equations.

Methodology: This cross-sectional study was conducted on 30 cases of invasive breast carcinoma. Immunohistochemical expression of ER, PR, HER2/neu, and Ki-67 was evaluated, and molecular subtypes were classified accordingly. Receptor concordance between primary tumors and corresponding metastatic deposits was assessed using McNemar’s test for paired categorical data. Magee recurrence scores were calculated to estimate recurrence risk. Descriptive statistics were used to summarize clinicopathological characteristics, and a p-value <0.05 was considered statistically significant.

Results: Thirty patients with invasive breast carcinoma were included, with a mean age of 44.8 ± 2.3 years. ER positivity was observed in 23 cases (76.7%), PR positivity in 15 cases (50.0%), and HER2/neu positivity in three cases (10.0%). Luminal A was the predominant molecular subtype, accounting for 22 (73.3%) cases. Among the 19 paired primary and metastatic samples, ER and PR discordance was identified in 14 (73.7%) and 10 (52.6%) cases, respectively, whereas no HER2/neu discordance was observed. Among cases with nodal metastasis, involvement of 1-3 lymph nodes was most frequent, occurring in 11 (57.9%) cases. One patient (5.3%) had distant brain metastasis. H-score analysis demonstrated that ER H-scores of 101-200 and 201-300 were observed in 12 (52.2%) and 10 (43.5%) cases, respectively, whereas PR H-scores of 101-200 and 201-300 were observed in six (40.0%) and eight (53.3%) cases, respectively. Based on aggregate Magee scores, 16 (53.3%) patients were categorized as low risk, nine (30.0%) as intermediate risk, and five (16.7%) as high risk.

Conclusion: Immunohistochemical profiling revealed a predominance of luminal A tumors and substantial discordance in ER and PR expression between primary tumors and metastatic deposits. Magee score assessment demonstrated that most patients belonged to the low-risk category, supporting its potential utility as a cost-effective prognostic tool in resource-limited settings.

## Introduction

Breast carcinoma is the most common malignancy among women worldwide and remains a leading cause of cancer-related mortality. In India, it has surpassed cervical cancer as the most frequently diagnosed cancer among women in several urban cancer registries [1]. The increasing incidence of breast carcinoma has been attributed to changes in lifestyle, reproductive patterns, and urbanization [1].

Breast carcinoma is a biologically heterogeneous disease with marked variations in clinical behavior, treatment response, and prognosis. Molecular classification based on the expression of estrogen receptor (ER), progesterone receptor (PR), human epidermal growth factor receptor 2 (HER2/neu), and Ki-67 categorizes tumors into surrogate molecular subtypes, including Luminal A, Luminal B, HER2-enriched, and triple-negative breast cancer (TNBC). These subtypes provide important prognostic information and influence therapeutic decision-making [2].

Although gene expression profiling remains the reference standard for molecular classification, its routine use is limited by cost and availability in many healthcare settings. Immunohistochemistry (IHC)-based surrogate classification provides a practical and clinically validated alternative. Hormone receptor-positive tumors are generally associated with favorable outcomes, whereas tumors with a high Ki-67 proliferation index often demonstrate more aggressive behavior and an increased risk of recurrence [3].

Changes in receptor expression between primary tumors and metastatic deposits may influence treatment selection and clinical outcomes. Therefore, reassessment of receptor status in metastatic disease has important therapeutic implications [3]. The Magee recurrence score, derived from routinely available histopathological and immunohistochemical parameters, has emerged as a potential surrogate tool for estimating recurrence risk and guiding treatment decisions in resource-constrained settings [4].

The present study aimed to evaluate the immunohistochemical expression of ER, PR, HER2/neu, and Ki-67 in invasive breast carcinoma; classify tumors into surrogate molecular subtypes based on immunohistochemical profiles; assess receptor concordance between paired primary breast carcinomas and their corresponding synchronous metastatic deposits, where available; and estimate recurrence risk using the Magee score equations. As a descriptive cross-sectional study, the objective of the Magee score assessment was to categorize the risk of pathological recurrence based on routinely available clinicopathological parameters rather than to evaluate patient outcomes or long-term prognosis.

## Materials and methods

Study design and setting

This cross-sectional observational study was conducted in the Department of Pathology, Gandhi Medical College and Hospital, Hyderabad, India, over a period of 24 months from May 2019 to May 2020. Ethical approval was obtained from the Institutional Ethics Committee prior to study initiation.

Study population and sample selection

Consecutive patients with histopathologically confirmed breast carcinoma during the study period and fulfilling the eligibility criteria were included. A total of 30 cases were enrolled. Demographic details, clinical history, operative findings, and relevant pathological data were retrieved from medical records and pathology requisition forms.

Female patients of any age with biopsy-proven or surgically resected breast carcinoma specimens that were adequately fixed in 10% neutral buffered formalin and suitable for histopathological and immunohistochemical evaluation were included. Cases with benign breast lesions, non-neoplastic conditions, autolysed specimens, extensively necrotic tissue, poorly preserved samples, or inadequate biopsy material were excluded.

Histopathological examination

All tissue specimens were fixed in 10% neutral buffered formalin, routinely processed, embedded in paraffin wax, and sectioned at 4-5 µm thickness. Sections were stained with hematoxylin and eosin (H&E) and examined under light microscopy.

Tumors were classified according to the World Health Organization (WHO) classification of breast tumors [5]. Histological grading was performed using the modified Bloom-Richardson grading system (Elston-Ellis modification) [6], based on tubule formation, nuclear pleomorphism, and mitotic count. Tumor size, lymph node status, and pathological stage were recorded according to the American Joint Committee on Cancer (AJCC) staging system, 8th edition [7].

Immunohistochemistry

Representative tumor blocks were selected for immunohistochemical analysis. Paraffin-embedded sections of 4-5 µm thickness were mounted on poly-L-lysine-coated slides. After deparaffinization and rehydration, antigen retrieval was performed using Tris-EDTA buffer (pH 9.0) in a microwave oven at 95°C for three cycles. Endogenous peroxidase activity was blocked using 3% hydrogen peroxide for 10 minutes.

Sections were incubated with primary antibodies against ER, PR, HER2/neu, and Ki-67 as per manufacturer instructions. Detection was performed using a polymer-based horseradish peroxidase system, followed by diaminobenzidine (DAB) chromogen development. Slides were counterstained with hematoxylin, dehydrated, cleared, and mounted.

The antibodies used included anti-ER rabbit monoclonal antibody (Biogenex), anti-PR rabbit monoclonal antibody clone EP2 (Biogenex), anti-HER2/neu mouse monoclonal antibody clone CB11 (Biogenex), and anti-Ki-67 mouse monoclonal antibody clone MIB-1 (Biogenex). Appropriate positive and negative controls were included in each run to ensure staining reliability and quality control.

Immunohistochemical scoring

ER and PR expression was evaluated using the Allred scoring system [8], incorporating both staining intensity and proportion of positive tumor cells. Tumors with an Allred score of ≥3 were considered positive.

HER2/neu expression was interpreted according to the American Society of Clinical Oncology/College of American Pathologists (ASCO/CAP) guidelines [9], where scores of 0 and 1+ were considered negative, 2+ equivocal, and 3+ positive.

The Ki-67 proliferation index was assessed by calculating the percentage of positively stained tumor nuclei in representative high-proliferation areas. Ki-67 expression was categorized as low (<10%), borderline (10-20%), and high (>20%) [10].

In addition, hormone receptor expression was quantified using the H-score method, generating values ranging from 0 to 300 based on staining intensity and percentage of positive cells [11].

Molecular subtyping

Tumors were classified into surrogate molecular subtypes based on immunohistochemical staining for ER, PR, HER2/neu, and Ki-67, according to the St. Gallen International Expert Consensus recommendations. The molecular subtypes included Luminal A, Luminal B, HER2-enriched, and TNBC.

Assessment of receptor concordance

Receptor concordance analysis was performed only in cases with paired primary breast carcinoma and corresponding metastatic tissue available for evaluation (n = 19). In most cases, the paired metastatic specimens consisted of synchronous axillary lymph node metastases obtained during surgical resection of the primary tumor. In contrast, one case had a distant brain metastasis. Immunohistochemical expression of ER, PR, HER2/neu, and Ki-67 was assessed in both paired specimens, and concordance or discordance was determined by direct comparison of receptor status according to the predefined immunohistochemical interpretation criteria for each biomarker.

Magee recurrence score assessment

The Magee recurrence score was assessed according to the published Magee score equations [12]. Patients were categorized into low-risk (<18), intermediate-risk (18-30), and high-risk (>30) groups based on the recorded Magee score assessment, and the distribution of cases across these risk categories was analyzed.

Statistical analysis

Statistical analysis was performed using IBM SPSS Statistics for Windows, Version 23 (Released 2015; IBM Corp., Armonk, New York, United States). Continuous variables were summarized as mean ± standard deviation, whereas categorical variables were presented as frequencies and percentages. Receptor concordance between primary tumors and corresponding metastatic deposits was evaluated using McNemar’s test for paired categorical data. Magee score-based risk categories were summarized as frequencies and percentages. A p-value of less than 0.05 was considered statistically significant.

## Results

Among the 30 study participants, the majority were aged ≥40 years (19, 63.3%), while 11 (36.7%) were younger than 40 years. The mean age was 44.8 ± 2.3 years. Grade II tumors were the most frequently observed histologic grade, accounting for 20 (66.7%) cases, followed by Grade I in seven (23.3%) and Grade III in three (10.0%) cases. Invasive ductal carcinoma, NST was the predominant histological subtype, comprising 26 (86.7%) cases, whereas invasive lobular carcinoma, medullary carcinoma, and invasive papillary carcinoma were relatively uncommon, representing one case (3.3%), two cases (6.7%), and one (3.3%) case, respectively. With respect to tumor size, T2 tumors (2-5 cm) were the most common, occurring in 15 (50.0%) patients, followed by T1 tumors (<2 cm) in 13 patients (43.3%) and T3 tumors (>5 cm) in two patients (6.7%) (Table 1).

**Table 1:** Baseline clinicopathological characteristics of breast carcinoma cancer

Variables	Total Number N (%)
Age <40 years	11 (36.7)
Age ≥40 years	19 (63.3)
Mean age (years)	44.8 ± 2.3
Histologic grade distribution (Modified Bloom–Richardson grading)	
Grade I — Well differentiated (score 3 to 5)	7 (23.3)
Grade II — Moderately differentiated (score 6 to 7)	20 (66.7)
Grade III — Poorly differentiated (score 8 to 9)	3 (10.0)
Histological type	
Invasive ductal carcinoma, NST (NOS)	26 (86.7)
Invasive lobular carcinoma	1 (3.3)
Medullary carcinoma	2 (6.7)
Invasive papillary carcinoma	1 (3.3)
Tumor size and stage	
< 2 cm (T1)	13 (43.3)
2–5 cm (T2)	15 (50.0)
> 5 cm (T3)	2 (6.7)

ER positivity was observed in 23 (76.7%) cases, while PR positivity was identified in 15 (50.0%) cases. HER2/neu positivity was present in three (10.0%) patients. Among the molecular subtypes, Luminal A was the predominant subtype, accounting for 22 (73.3%) cases, followed by TNBC in five cases (16.7%), HER2-enriched in two cases (6.7%), and Luminal B in one case (3.3%). Evaluation of the Ki-67 index showed that low expression (<10%) was the most common finding, observed in 16 (53.3%) cases, whereas borderline (10-20%) and high (>20%) expression were seen in 10 (33.3%) and four (13.3%) cases, respectively (Table 2).

**Table 2:** Immunohistochemical profile and molecular subtypes IHC: Immunohistochemistry; HER2: human epidermal growth factor receptor 2

Variables	Total Number N (%)
IHC markers	
Positive estrogen receptor (ER)	23 (76.7%)
Positive progesterone receptor (PR)	15 (50%)
Positive HER2/neu	3 (10%)
Molecular subtype	
Luminal A	22 (73.3%)
Luminal B	1 (3.3%)
HER2-enriched	2 (6.7%)
Triple-negative breast cancer (TNBC)	5 (16.7%)
Ki-67 index	
< 10% (low)	16 (53.3%)
10–20% (borderline)	10 (33.3%)
> 20% (high)	4 (13.3%)

Among the 19 cases with metastatic deposits, involvement of 1-3 lymph nodes constituted the most frequent nodal burden category and was observed in 11 (57.9%) cases, followed by involvement of 4-7 lymph nodes in seven (36.8%) cases and involvement of ≥8 lymph nodes in one (5.3%) case. Distant brain metastasis was identified in one (5.3%) patient (Table 3).

**Table 3:** Nodal burden and brain metastasis among metastatic cases (n = 19)

Variables	No. of Cases	%
Nodal burden		
1–3 nodes	11	57.9%
4–7 nodes	7	36.8%
≥ 8 nodes	1	5.3%
Brain metastasis (distant)	1	5.3%

Among the 19 paired primary and secondary tumor samples, ER discordance was observed in 14 (73.7%) cases and PR discordance in 10 (52.6%) cases. No discordance was identified for HER2/neu status, whereas Ki-67 discordance was present in six (31.6%) cases. McNemar’s test demonstrated significant discordance for both ER (p = 0.001) and PR (p = 0.046), indicating a significant change in receptor expression between primary tumors and secondary deposits (Table 4).

**Table 4:** IHC receptor discordance between primary tumors and secondary deposits (n=19 paired cases) ER: Estrogen receptor; PR: progesterone receptor; IHC: immunohistochemistry; HER2: human epidermal growth factor receptor 2

Marker	Positive	Negative	Discordant (n)	Discordance %	McNemar’s Test	p-value
ER	2	17	14	73.7%	10.24	0.001
PR	6	13	10	52.6%	4	0.046
HER2/neu	2	17	0	0%	-	-
Ki-67	—	—	6	31.6%	-	-

Among the 23 ER-positive cases, the majority demonstrated H-scores between 101-200 and 201-300, accounting for 12 (52.2%) and 10 (43.5%) cases, respectively, while only one (4.3%) case had an H-score between 1 and 100. Similarly, among the 15 PR-positive cases, H-scores of 201-300 were most common, observed in 8 (53.3%) cases, followed by scores of 101-200 in 6 (40%) cases. Only one (6.7%) PR-positive case exhibited an H-score between 1 and 100. No cases showed an H-score of <1 for either marker, indicating that most receptor-positive tumors demonstrated moderate to strong expression levels (Table 5).

**Table 5:** H-score distributions for ER and PR ER: Estrogen receptor; PR: progesterone receptor

H-Score Range	ER+ Cases	% (of 23)	PR+ Cases	% (of 15)
< 1	0	0%	0	0%
1–100	1	4.3%	1	6.7%
101–200	12	52.2%	6	40%
201–300	10	43.5%	8	53.3%

Magee recurrence score assessment revealed that 16 (53.3%) patients were classified as low risk, 9 (30.0%) as intermediate risk, and 5 (16.7%) as high risk. Low-risk scores constituted the largest proportion of cases, whereas high-risk scores were observed in a relatively smaller subset of patients (Table 6).

**Table 6:** Magee recurrence score distribution (n=30)

Aggregate Magee score	No. of Cases	%
< 18 (low risk)	16	53.3%
18–30 (intermediate risk)	9	30.0%
> 30 (high risk)	5	16.7%

## Discussion

The mean age at presentation in the present study was 44.8 ± 2.3 years, which is lower than that reported in most international and Indian studies. Bhargava et al. [4] observed a median age of around 50 years, and Suman et al. [13] reported a mean age of 49 years. Large registry-based data from the National Cancer Registry Program [14] also report an average presentation age of 50-53 years. Compared to these studies, the present cohort demonstrates a relatively younger age at diagnosis, supporting findings by Agarwal and Ramakant [1], who highlighted earlier onset of breast cancer in Indian populations due to genetic, reproductive, and healthcare-related factors.

In the present study, Grade II tumors were most frequent (66.7%), consistent with findings by Davis et al. [15], who reported Grade II as the predominant histological grade in clinical trial populations. This suggests a tumor biology pattern in the present population comparable to previously published studies with moderate-grade predominance. Invasive ductal carcinoma, no special type (NST), accounted for 86.7% of cases in the present study, aligning with global epidemiological observations described by Leong and Zhuang [16], who identified IDC-NST as the most common histological subtype worldwide. This is further supported by Feng et al. [2], who reported ductal carcinoma as the dominant histological form across diverse populations.

Luminal A was the most common molecular subtype in the present study (73.3%), which is higher than most published literature. This finding is closest to the results of Cheng et al. [17], who also reported Luminal A predominance, although at a lower proportion. The higher frequency in the present cohort may reflect the hormone receptor-rich biology of tumors in this population. TNBC accounted for 16.7% of cases, which is comparable to frequencies reported in similar population-based studies such as that by Fourati et al. [18]. At the same time, Mehdi et al. [19] also reported comparable TNBC proportions in regional cohorts. The relatively lower proportion of HER2-enriched and Luminal B subtypes may reflect sample size limitations and intrinsic tumor biology variation.

A major finding of this study was ER and PR discordance between paired primary breast tumors and their corresponding synchronous metastatic deposits. Although these discordance rates were higher than those reported in most published studies, the findings should be interpreted with caution given the limited sample size and cross-sectional design. Methodological factors, including pre-analytical and analytical variability inherent to immunohistochemical assessment, may also have contributed to the observed discordance. Therefore, while the findings suggest the presence of receptor heterogeneity between paired specimens, they should not be interpreted as definitive evidence of biological receptor instability. Larger prospective studies using standardized methodology are required to validate these observations. Zhao et al. [20] reported ER discordance of 27.8%, while lower rates have been observed in other series. Similarly, PR discordance in the present study (52.6%) was also higher than previously reported literature, including that by Zhao et al. [20]. The observed ER and PR discordance between paired primary breast tumors and their corresponding synchronous metastatic deposits suggests the presence of spatial intratumoral heterogeneity. Because the paired specimens were obtained during the same disease episode in this cross-sectional study, these findings should not be interpreted as evidence of temporal receptor conversion during disease progression or recurrence. Instead, they highlight the biological variability that may exist between primary tumors and synchronous metastatic sites.

In contrast, HER2/neu showed complete concordance (0%) between primary and metastatic sites, which is consistent with findings reported by Gonzalez-Angulo et al. [21], supporting the relative stability of HER2 expression during disease progression. Overall receptor variability observed in ER and PR, compared to HER2 stability, reflects the known differential behavior of biomarkers in breast carcinoma progression. This phenomenon is further supported by the broader concept of tumor evolution and biomarker instability described by Lindström et al. [22], who demonstrated that hormone receptors are more prone to temporal and spatial variation due to clonal evolution and therapeutic selection pressure.

Ki-67 expression demonstrated expected subtype-specific variation, with the lowest levels observed in Luminal A tumors (mean 8.9%) and the highest in triple-negative breast cancer (mean 32.2%), consistent with established literature. Ki-67 is a recognized marker of cellular proliferation and tumor aggressiveness. In the present study, Ki-67 discordance (31.6%) was lower than ER and PR discordance, suggesting relative preservation of proliferative activity between primary and metastatic deposits.

More than half of the cases (53.3%) had Magee scores below 18, suggesting low recurrence risk and potential omission of Oncotype DX testing in these patients. This finding is supported by Turner et al. [23], who demonstrated that modified Magee equations can reliably predict low-risk Oncotype DX categories. Similarly, Bhargava et al. [4] reported that low Magee scores correlate strongly with low recurrence scores and may obviate the need for genomic testing in selected cases. Additional validation by Sughayer et al. [24] and Hou et al. [25] has demonstrated strong concordance between Magee-derived scores and Oncotype DX recurrence risk categories, supporting their utility as a cost-effective triage tool in resource-limited settings.

Limitations

This study has several limitations. First, it was conducted at a single tertiary care center with a relatively small sample size of 30 cases, which may limit the generalizability of the findings to broader populations. Second, molecular classification was based on immunohistochemical surrogate markers rather than gene-expression profiling techniques, which are considered the gold standard for intrinsic subtype characterization. Third, receptor concordance analysis was performed only in cases with available metastatic deposits, resulting in a limited number of paired samples for comparison. Fourth, long-term clinical follow-up data, including disease-free survival, overall survival, and treatment response, were not available; therefore, the prognostic significance of receptor discordance and Magee recurrence scores could not be evaluated. Additionally, although patients were categorized into standard Magee recurrence risk groups, the individual numerical Magee scores were unavailable for further correlation analyses with clinicopathological parameters. Consequently, assessment of the relationship between absolute Magee scores and individual pathological variables could not be performed. Future studies incorporating complete numerical Magee score data may provide further insights into its prognostic utility. Finally, direct comparison of Magee scores with commercial multigene assays such as Oncotype DX was not performed because of resource constraints. Larger multicenter studies incorporating genomic profiling and long-term outcome assessment are warranted to validate these findings.

## Conclusions

This study demonstrates IHC-based molecular subtyping in a South Indian breast carcinoma cohort presenting at a younger age than that described in Western literature. Luminal A was the predominant molecular subtype. High ER and PR discordance between paired primary breast tumors and synchronous metastatic deposits highlights the presence of receptor heterogeneity between anatomically distinct tumor sites. These findings support consideration of receptor assessment in metastatic tissue when clinically appropriate. However, owing to the cross-sectional design, the present study does not permit conclusions regarding temporal receptor conversion or changes occurring during disease progression or recurrence. The findings also suggest that Magee score-based risk stratification may represent a practical adjunct for estimating recurrence risk in resource-limited settings. However, because this study was descriptive and lacked clinical follow-up or direct comparison with genomic assays, these observations should be considered hypothesis-generating and require validation in larger prospective studies.
